# Medical Registry Data Collection Efficiency: A Crossover Study Comparing Web-Based Electronic Data Capture and a Standard Spreadsheet

**DOI:** 10.2196/jmir.5576

**Published:** 2016-06-08

**Authors:** Pedro Vinícius Staziaki, Phillip Kim, Harshna V Vadvala, Brian B Ghoshhajra

**Affiliations:** ^1^Massachusetts General HospitalDepartment of RadiologyHarvard Medical SchoolBoston, MAUnited States

**Keywords:** electronic data capture, clinical research, translational research, registry, data management

## Abstract

**Background:**

Electronic medical records and electronic data capture (EDC) have changed data collection in clinical and translational research. However, spreadsheet programs, such as Microsoft Excel, are still used as data repository to record and organize patient data for research.

**Objective:**

The objective of this study is to assess the efficiency of EDC as against a standard spreadsheet in regards to time to collect data and data accuracy, measured in number of errors after adjudication.

**Methods:**

This was a crossover study comparing the time to collect data in minutes between EDC and a spreadsheet. The EDC tool used was Research Electronic Data Capture (REDCap), whereas the spreadsheet was Microsoft Excel. The data collected was part of a registry of patients who underwent coronary computed tomography angiography in the emergency setting. Two data collectors with the same experience went over the same patients and collected relevant data on a case report form identical to the one used in our Emergency Department (ED) registry. Data collection tool was switched after the patient that represented half the cohort. For this, the patient cohort was exactly 30 days of our ED coronary Computed Tomography Angiography registry and the point of crossover was determined beforehand to be 15 days. We measured the number of patients admitted, and time to collect data. Accuracy was defined as absence of blank fields and errors, and was assessed by comparing data between data collectors and counting every time the data differed. Statistical analysis was made using paired *t* -test.

**Results:**

The study included 61 patients (122 observations) and 55 variables. The crossover occurred after the 30th patient. Mean time to collect data using EDC in minutes was 6.2±2.3, whereas using Excel was 8.0±2.0 (*P* <.001), a difference of 1.8 minutes between both means (22%). The cohort was evenly distributed with 3 admissions in the first half of the crossover and 4 in the second half. We saw 2 (<0.1%) continuous variable typos in the spreadsheet that a single data collector made. There were no blank fields. The data collection tools showed no differences in accuracy of data on comparison.

**Conclusions:**

Data collection for our registry with an EDC tool was faster than using a spreadsheet, which in turn allowed more efficient follow-up of cases.

## Introduction

Electronic medical records and electronic data capture (EDC) have changed data collection in clinical and translational research [1]. Electronic forms reduce inaccurate data entry and study costs because the data are entered directly into an electronic form on a computer [2]. However, spreadsheet programs are still used as data repository to record and organize patient data for research. This method of data storage is not only limited in the organization and quality of data but also increases the likelihood of incorrect data entry [3].

It is known that EDC reduces cost and time when compared with paper-based data collection [4-6]. However, there is little research about how EDC solutions are better compare with spreadsheets. Furthermore, most of the literature about EDC is descriptive, focusing only on the technology, the methods, or the experience [7].

The objective of this study was to assess the efficiency of an EDC solution compared with a standard spreadsheet regarding time to collect data and data accuracy, measured in number of errors after adjudication. We hypothesized that EDC reduces the time of data collection without compromising accuracy, as compared with a standard spreadsheet.

## Methods

This was a single-institution crossover study comparing the time to collect data in minutes between an EDC tool and a spreadsheet. This study was approved by the Institutional Review Board and was Health Insurance Portability and Accountability Act (HIPAA) compliant.

### Study Design

**Figure 1:**
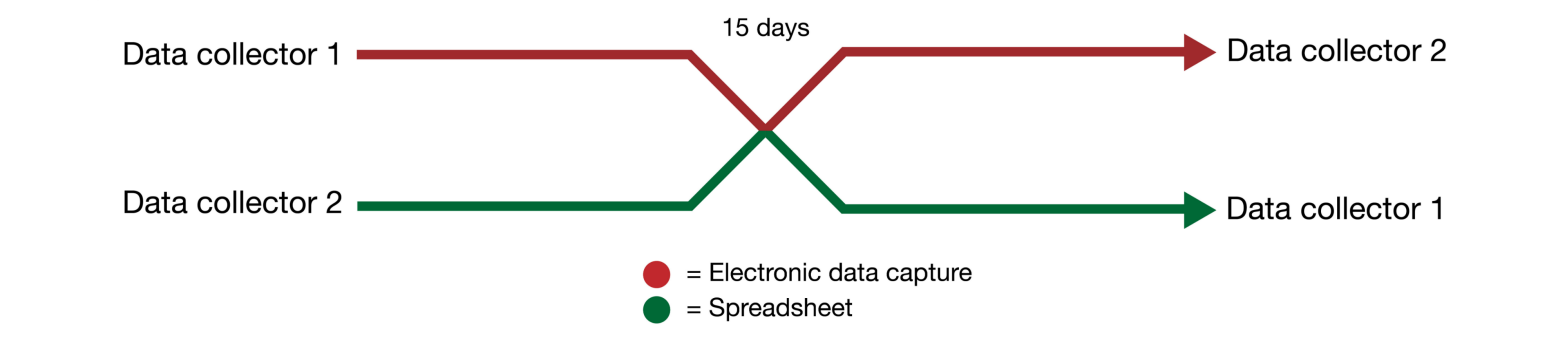
Crossover design.

Two data collectors (“1” and “2”) went over the same patients and collected relevant clinical and imaging data, switching data collection tool after the patient that represented half the cohort (Figure 1). Both data collectors observed each patient, one collecting data on EDC and other on a spreadsheet.

**Figure 2:**
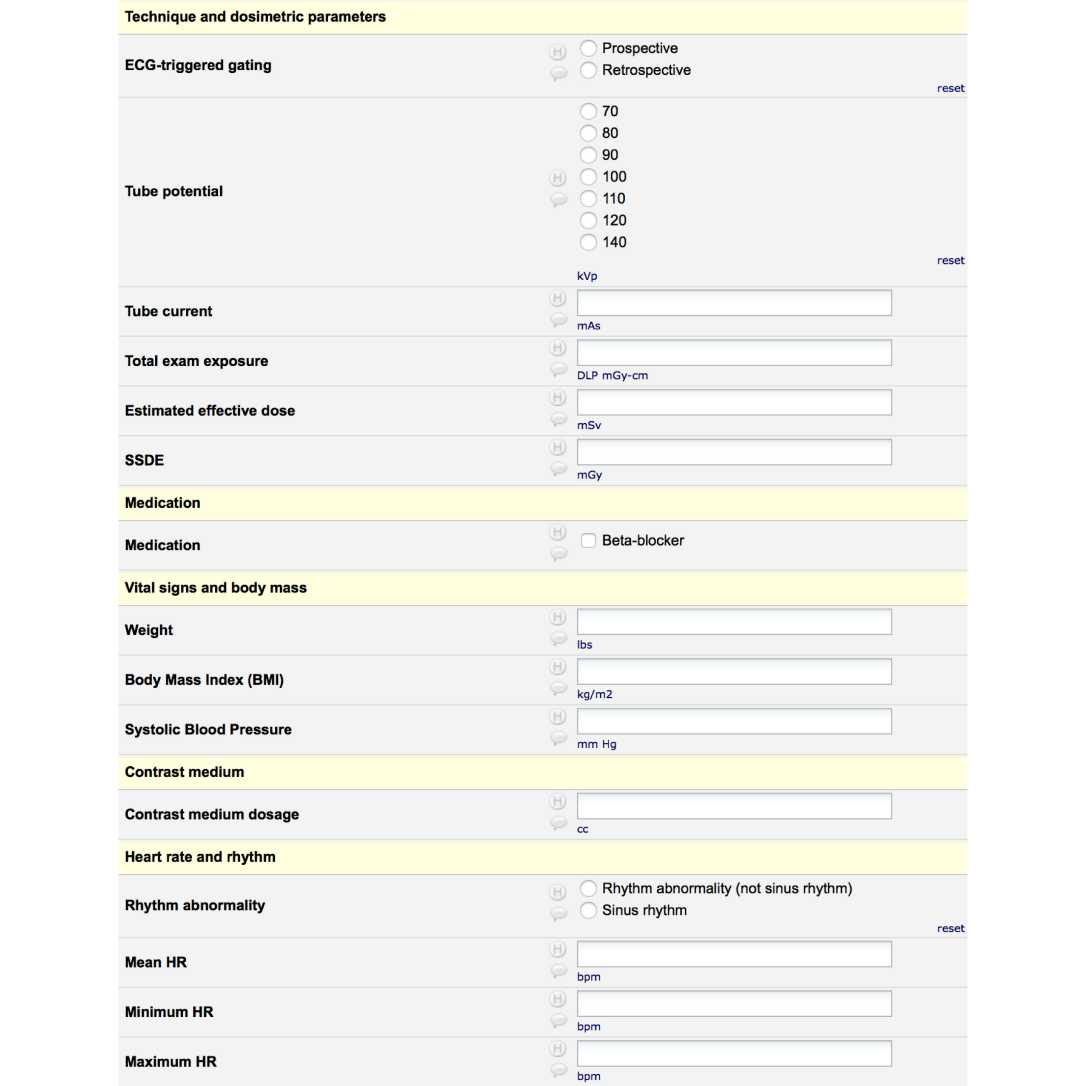
A sample of our ED registry case report form (CRF).

We designed this study to simulate the actual registry data collection environment. For this, the patient cohort was exactly 30 days of our Emergency Department (ED) coronary Computed Tomography Angiography registry and the point of crossover was determined beforehand to be 15 days. The case report form (CRF) for this study was the same as used in our ED registry (Figure 2).

Anticipating that certain patients would be admitted to the hospital and contain more data to be collected, we also looked at how many of those patients were admitted, in order to know if they were evenly distributed between each half of the crossover.

The EDC tool used was Research Electronic Data Capture (REDCap) [8] and the spreadsheet application was Excel (Microsoft Corporation, Redmond, Washington).

Each data collectors had 5 months’ experience in registry data collection and used the same versions of REDCap and Excel and an electronic medical record system (QPID). Both users worked on the same computer systems having the same Internet speed. The CRFs on each data collection tool had identical variables, which comprised dichotomous variables, categorical variables, and continuous variables.

**Figure 3:**
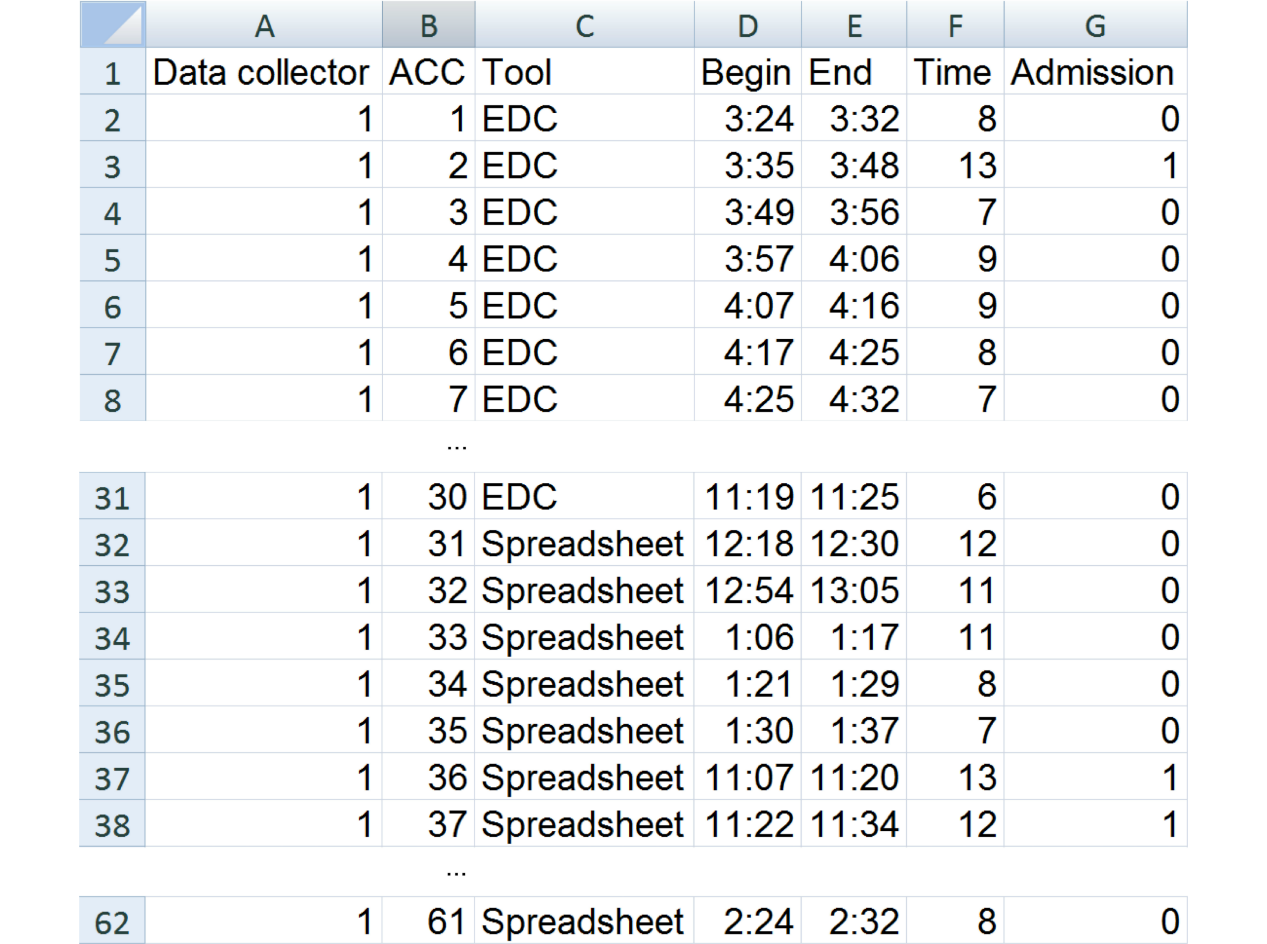
Sample of the first rows of time data for data collector “1”, for each data collection tool. The full cohort contained 61 rows. Column A identifies the data collector (“1” or “2”). Column B identifies the study subject (each registry record) with their accession number (ACC), here anonymized. Column C indicates the tool used to collect the registry data. Column D indicates the time-stamp of the start of data collection, whereas column E indicates the end. Column F contains the calculated time of data collection in minutes (column E minus column D). Admission was coded in column G as 1 for presence and 0 for absence. EDC = Electronic data capture.

The time to collect data was recorded in a separate spreadsheet (Figure 3). Both data collectors recorded time identically irrespective of the tool (spreadsheet or EDC) used by them for the registry data collection. This spreadsheet was different from the data collection spreadsheet that was to be compared with EDC.

### Time and Accuracy Data

In order to collect the time data, we manually typed the time stamp of the beginning of data collection in one column and the end of data collection in another column in the time spreadsheet. Time was calculated in minutes by subtracting the beginning time-stamp from the end time-stamp and it was recorded in the next column.

Accuracy was assessed by comparing three indicators between both data collection tools: the number of blank fields, the number of discrepant fields, and the type of discrepancies. For the type of discrepancy, we looked at every pair of record, comparing a record from one tool to the same record from the other tool.

The discrepancies were categorized into two groups: different content and same content errors, such as typos. Discrepancies that represented different content were adjudicated by a senior radiologist to select which record in each pair is deemed the wrong data entry.

### Statistical Analysis

Statistical analysis was made using paired *t* -test. Every patient was tested twice, as each was collected once on REDCap and once on spreadsheet.

## Results

The study included 61 patients (122 observations) and 55 variables. The crossover occurred after the 30th patient. Mean time to collect data using EDC in minutes was 6.2±2.3, whereas using a spreadsheet was 8.0±2.0 (*P* <.001), resulting in a reduction of 1.8 out of 8 minutes (22%). The cohort was evenly distributed, with 3 admissions in the first half of the crossover and 4 in the second half.

In all, 6710 entries of the registry were collected (61 patients × 55 variables, 2 collectors). We saw 2 continuous variable typos out of 6710 (<0.1 %) that a single data collector made in Excel. There were no blank fields and no discrepancies.

## Discussion

The main finding of this study was that less time is required to collect data to an EDC than to a spreadsheet. Prior literature has compared EDC with conventional paper capture methods and it is mainly descriptive. This study compared objectively the time to collect data between a Web-based rigid form and a standard spreadsheet, and confirmed that EDC using REDCap can be more time effective. We chose to compare EDC to spreadsheets since we have found that in the era of electronic medical records, efficiency can be gained by using only EDC, and the final form of data delivered for research analysis is usually always electronic.

Regarding the time to collect a single data endpoint, a small difference in time can add up to a significant difference in the long term. It took 6.3 h to collect the data in REDCap compared with 8.1 hin Excel, a difference of 1.8 h. In our clinical registry of over 1000 ED admissions, this means that by collecting all data via this EDC solution we would spend only 103 h (6.2 min × 1000 observations) as opposed to 133 (8.0 × 1000), saving more than 3 workdays of data collection.

Concerning accuracy, there were no discrepancies between the two data collection tools. The number of errors was too small compared with the number of observations collected. Due to this, we did not perform a statistical analysis of the number of errors in data entry. In addition, since single data collector made typos in the spreadsheet, we did not see differences in data collection that could be attributed to a specific tool in our study.

Setting up ranges and automatic calculations can prevent these errors. Range checks make sure the collector does not insert a typo that would give a value in continuous variables that would not make sense [9]. While these can be set up in both Excel and REDCap, the latter can provide a better interface making it is easier to be done.

Many EDC tools have been analyzed [8,10-12]. The major advantage of electronic CRFs over spreadsheets is that the former can be designed to present only certain acceptable choices for an item or to check the syntax and range of data that are entered [9]. This reduces the likelihood of data entry errors.

REDCap was developed at Vanderbilt University’s Institute for Clinical and Translational Research for building and managing online databases [8]. REDCap is an NIH-sponsored, HIPAA-compliant, noncommercial, and secure EDC solution. It supports retrospective and prospective studies, as well as multicenter clinical trials. It has an intuitive user-friendly interface for data entry, allowing researchers to create secure online forms with very large numbers and several types of variables and does not require any technical skillset to implement. By organizing the variables into forms, it also permits the user to save the progress between each form, avoiding the dramatic trouble of losing data by not saving the progress. These forms also allow for mid-study modifications without affecting previously collected data.

Data collections in standard spreadsheets can be easily imported to REDCap, and then data can be exported into most major statistical software packages, such as Stata (StataCorp, College Station, TX, USA), SAS (SAS Institute, Cary, NC, USA), R (R Foundation for Statistical Computing, Vienna, Austria), and SPSS (IBM Corporation, Armonk, New York), as well as comma-delimited files. As it is a Web-based tool, it is compatible with all operating systems [13] and requires no installation of software [14].

Irrespective of the data collection tool used, the data is often exported to a comma-delimited file that can be read as a spreadsheet. Then, this file can be imported into statistical software packages. Moreover, spreadsheets are the common file format through which researchers and statisticians exchange the data.

However, spreadsheets require the data collector to abide by certain practices regarding how data are organized and formatted within the spreadsheet [15], such as putting variable names in a single row and avoiding the use of special characters. Also, spreadsheets for data collection restricts features such as colored text, cell shading, commas, merging cells, comments, or mixing data types in a single column. This makes the data collection more time-consuming and error-prone.

This study has limitations. A limitation of our study is that it is a small dataset.

In addition, the times to collect data reported are inherent to our registry, and would be different in different research studies. Therefore, it is difficult to extrapolate our results to other research projects. Nevertheless, the use of a crossover design ensured the data was controlled, and this method accounted for differences in speed inherent to each collector.

In conclusion, data collection for our registry with EDC was faster than using a spreadsheet, allowing more efficient follow-up of cases.

## Abbreviations

CRF: case report form
ED: emergency department
EDC: electronic data capture
